# Reconstruction of the Vulva and Perineum—Comparison of Surgical Methods

**DOI:** 10.3390/jcm14134456

**Published:** 2025-06-23

**Authors:** Anna Jędrasiak, Honorata Juniewicz, Wiktoria Raczek, Alicja Srokowska, Mateusz Kozłowski, Aneta Cymbaluk-Płoska

**Affiliations:** Department of Reconstructive Surgery and Gynecological Oncology, Pomeranian Medical University in Szczecin, Al. Powstańców Wielkopolskich 72, 70-111 Szczecin, Poland; honorataj96@gmail.com (H.J.); wiktoriaraczek@gmail.com (W.R.); alicjasrokowska112@gmail.com (A.S.); mtkoozo@gmail.com (M.K.); aneta.cymbaluk@gmail.com (A.C.-P.)

**Keywords:** vulvar and perineal reconstruction, reconstructive surgery, vulvar cancer, surgical treatment, oncological surgery

## Abstract

Vulvar and perineal reconstruction represents a significant surgical challenge, particularly in the context of oncological resections, trauma, or postpartum injuries. Vulvar cancer, predominantly squamous cell carcinoma, often necessitates extensive resections, leading to significant tissue defects and high rates of postoperative complications. Reconstructive procedures, encompassing skin grafts and local, regional, and distant flaps, are intended to restore the anatomical structure, protect internal organs, and enhance functional outcomes and quality of life. The selection of technique is contingent upon the dimensions of the defect, its location, the patient’s condition, and the availability of suitable tissue. The management of minor defects can be accomplished through local advancements, while more extensive or complex cases necessitate the utilization of musculocutaneous (e.g., VRAM) or fasciocutaneous (e.g., ALT) flaps. The present article provides a review of surgical methods of vulvar and perineal reconstruction, with a focus on indications, techniques, and potential complications.

## 1. Introduction

The reconstruction of the vulva and perineum represents a significant challenge in gynecological surgery, particularly in cases of deformity, trauma, or defects resulting from long-term medical treatment. Typically, these surgical interventions are performed for serious conditions such as tumors, postpartum injuries, or perianal fistulas, with the potential for significant anatomical and functional complications. A fundamental component of the management of patients afflicted with genital cancers, particularly vulvar cancer, perineal reconstruction following cancer surgery is of paramount importance. While vulvar cancer accounts for approximately 3–5% of all malignancies within the female genital tract, recent epidemiological studies from 13 high-income countries indicate that this percentage may be increasing, revealing a significant overall increase in the incidence of vulvar cancer of 14% [1,2]. Concurrent with this trend, there has been an increase in the incidence of vulvar intraepithelial neoplasia (VIN), a precursor lesion of vulvar cancer. A nationwide Dutch registry-based study reported that between 1991 and 2011, the European standardized incidence rate (ESR) of high-grade squamous intraepithelial lesion (HSIL) increased from 2.39 to 3.26 per 100,000 woman-years, while the ESR of differentiated VIN (dVIN) rose from 0.02 to 0.08 per 100,000 woman-years. Of particular significance is the finding that the 10-year cumulative risk of progression to vulvar squamous cell carcinoma (VSCC) was estimated at 9.7% for HSIL and as high as 50.0% for dVIN, thereby underscoring the aggressive potential of the latter and the necessity for early identification and appropriate treatment strategies [3]. The management of VIN typically involves surgical excision. Nevertheless, recurrence remains a frequent occurrence. A retrospective study involving 784 women found a recurrence rate of 26.3%, with 2.2% progressing to invasive cancer. Risk factors for recurrence included an age over 50, immunosuppression, positive surgical margins, and a coexisting HPV infection or lichen sclerosis [4]. In particular, dVIN has been shown to be associated with a significantly higher risk of malignant transformation and is often underdiagnosed. Its clinical and histological features are subtle, and the morphological spectrum is broader than is commonly appreciated. These diagnostic challenges frequently result in delayed treatment or incomplete resection, thereby increasing the likelihood of multiple surgical interventions [5]. In a recent cohort study of 76 patients with dVIN, the absolute risk of progression to VSCC after solitary dVIN excision was 43.2%. In the same study, the recurrence rate of VSCC in patients with coexisting dVIN-VSCC was 31.3% [6]. These findings have direct implications for surgical planning. The presence of extensive or recurrent VIN, especially dVIN, frequently necessitates repeated excisions and may result in more complex perineal reconstruction procedures. The most prevalent form of vulvar cancer is squamous cell carcinoma (SCC), accounting for over 90% of all cases [7]. Treatment of vulvar cancer is chiefly dependent on surgical intervention. In instances where resection is not a viable option, radiation therapy is recommended as a treatment modality. In cases of recurrence or advanced disease, chemotherapy is employed [8]. International guidelines emphasize the necessity of individualizing therapy, combining oncological control with quality of life [9]. The surgical treatment of vulvar cancer often necessitates extensive resection, resulting in significant tissue loss in the perineum. Furthermore, this malignancy carries a significant risk of local recurrence, even following wide excision [10]. Despite advancements in techniques, aggressive resections remain associated with elevated rates of treatment-related morbidity and mortality. Postoperative wound complications represent a significant challenge in the management of these patients, with incidence rates ranging from 26% to 85% [11]. These complications are associated with significant physical and psychosocial problems, hospital readmissions, and increased healthcare costs [12]. Comprehensive epidemiological data on the incidence of perineal reconstruction after oncological surgery are lacking.

Reconstructive techniques vary and may include the use of skin grafts, local skin flaps, regional skin flaps, and distant skin flaps. The main aim of these reconstructive procedures after vulvar and perineal resection surgery is to restore the anatomical structure and function of these areas, thereby significantly improving the patient’s quality of life [13]. In addition, these techniques effectively close tissue defects while protecting internal organs and promoting the healing process. The appropriate reconstructive method should be selected based on both the location of the lesion on the vulva and the established diagnosis. Small defects should be repaired with simple skin advancements, such as V-Y advancement flaps. More extensive defects require advanced techniques, such as musculocutaneous flaps (e.g., VRAM) or fasciocutaneous flaps (e.g., ALT flap) [14]. For patients with a significantly limited tolerance for prolonged procedures, less invasive techniques, such as gracilis flaps, are recommended. Defects involving the urethra or rectum require VRAM flaps, as these provide reliable coverage for deep tissue losses [10]. The selection of a reconstructive technique should be based on an individualized assessment of the patient’s needs, including the extent of the defect, the patient’s tolerance for medical procedures, and the availability of suitable tissue.

To ensure the reliability and scientific integrity of the publications, the selection of publications was conducted according to clearly defined criteria. The analysis encompassed a comprehensive range of articles addressing various facets of vulvar cancer, including its epidemiology, treatment regimens, international guidelines, surgical techniques, postoperative complications, quality of life, and gynecological care. The present study encompasses publications containing original data, systematic reviews, and meta-analyses that were published between 1979 and 2025. The focus of this study is particularly on the last decade. Case reports lacking broader clinical significance and works comprising the replication of data without new analyses were excluded from the study. The relevant literature was selected from the PubMed database, with the selection process undertaken based on the analysis of titles and abstracts. The following keywords were utilized in the search: vulvar reconstruction, vulvar cancer, oncologic resection, perineal flap, keystone flap, VRAM, psychological impact of gynecologic surgery, quality of life in gynecologic cancer. The final selection comprised 96 publications that most accurately reflected the complexity of vulvar cancer treatment—from surgical, oncological, reconstructive, and psychological perspectives.

The aim of this article is to provide a detailed overview of the surficial methods used in vulvar and perineal reconstruction, focusing on their indications, operative techniques, and outcomes. Special attention is given to the description of surgical procedures, postoperative management, and potential complications.

## 2. Surgical Methods for Perineal and Vulvar Reconstruction

Perineal and vulvar reconstruction is a critical component of post-oncological treatment, particularly for patients with malignancies in the pelvic region. The choice of method depends on the extent of the defect and poses significant challenges due to the complex anatomy and the functional and aesthetic requirements of the patient [15].

A review of the literature provides extensive insights into reconstruction techniques, which can be categorized as regional, local, or distant approaches, while addressing specific approaches for individual clinical cases.

### 2.1. Sensate Gluteal Fold Flaps

The Sensate Gluteal Fold Flap technique is recommended as a primary method of reconstruction following the excision of vulvar malignancies such as melanoma, squamous cell carcinoma, or Paget’s disease in cases of reconstruction after radical vulvectomy [16]. It is also utilized in reconstructive surgeries following the resection of anorectal tumors [17]. The Sensate Gluteal Fold Flap is employed in reconstructions after total pelvic exenteration and serves as an alternative to rectus abdominis musculocutaneous flaps, particularly in patients with prior abdominal surgeries or stomas [18].

Gluteal fold flaps use tissue from the lower buttocks, offering excellent anatomical compatibility with the perineal region. During the flap’s preparation, attention is paid to preserving sensory nerves and blood vessels to ensure functionality and sensation in the reconstructed area. The procedure involves identifying perforating vessels using imaging studies or intraoperatively, preparing the flap from the gluteal fold area while maintaining the nerves and vessels, and transferring the flap to the defect site. The scar is concealed within the natural gluteal fold, enhancing cosmetic outcomes [16].

Anatomical studies indicate that the vessels supplying the flap are predictable, allowing for the adjustment of the flap’s volume by appropriately shaping the adipose tissue while preserving the nourishing vessels [19]. The preservation of sensory function is possible due to the use of an innervated flap, enabling patients to retain sensation in the reconstructed area, which is crucial for sexual function and overall patient comfort. An additional advantage of this method is that the reconstruction utilizes the patient’s own tissues, resulting in a more natural appearance of the perineal region [16].

**Figure 1:**
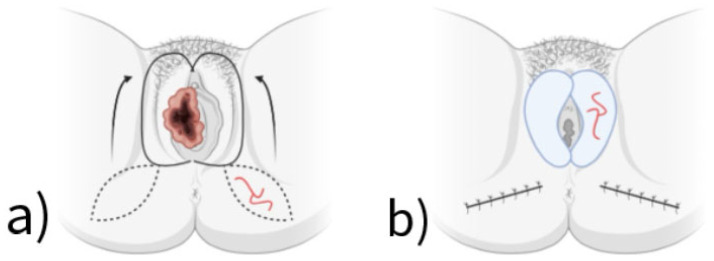
The illustration for the Sensate Gluteal Fold Flaps method includes (**a**) the location of the gluteal fold flaps from the lower portion of the buttocks; (**b**) the transfer of the flaps to the defect area in the perineal/vulvar region.

The use of the gluteal fold flap is a relatively simple technique associated with a low complication rate and favorable functional outcomes, ensuring the high success rate of the method [20]. A schematic visualization of the method is shown in Figure 1.

### 2.2. Triple Flap Technique

The Triple Flap Technique is recommended for the reconstruction of extensive defects resulting from the treatment of vulvar cancer, in cases where a single flap is insufficient to achieve complete coverage. The reconstruction is accomplished using bilateral V-Y advancement flaps from the gluteal folds in combination with a flap harvested from the mons pubis region [21]. This technique is also applicable in reconstructions following resection for Paget’s disease, where a musculocutaneous flap from the gracilis muscle, a V-Y advancement flap from the lower abdominal surface, and an island flap based on the internal pudendal artery perforator are utilized. This approach provides adequate coverage and preserves the functional integrity of the perineal region [22]. The Triple Flap Technique is additionally employed in reconstructive procedures for congenital anorectal malformations in infants. By combining three cutaneous flaps, the anal canal is reconstructed, enabling an appropriate sphincter tension and minimizing the risk of complications such as prolapse or anal stenosis [23].

**Figure 2:**
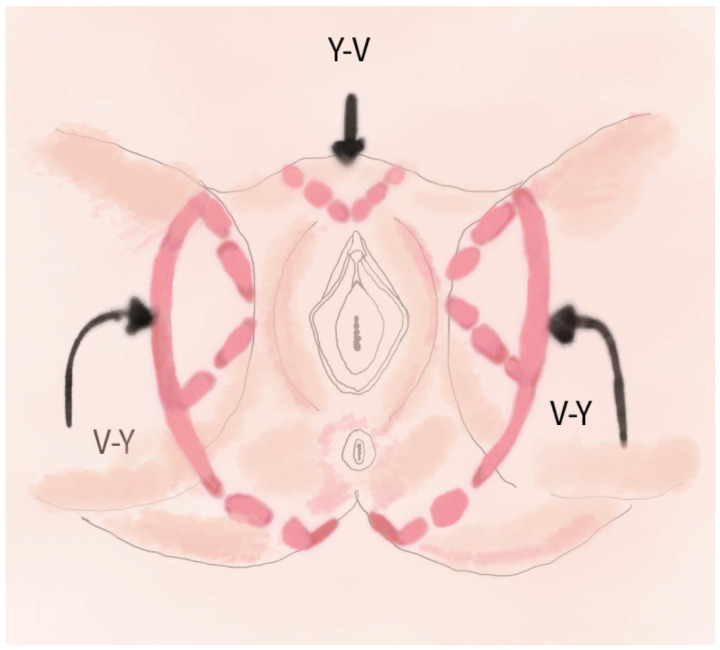
Illustration depicting the method of perineal reconstruction using two V-Y advancement flaps from the intragluteal fold region and a third Y-V flap from the mons pubis area: Two V-Y flaps from the intragluteal folds—drawn as triangular “wings”. One Y-V flap from the mons pubis area—drawn above the defect. Arrows indicate the direction of flap advancement toward the center.

This method involves harvesting three skin flaps to close both superficial and deep defects while minimizing tension on the wound edges. The technique involves making three incisions around the defect, preserving the vessels, mobilizing each flap for repositioning, and connecting the edges to create a unified reconstructive surface [21]. Two V-Y flaps are derived from the subgluteal fold region and are based on the perforators of the pudendal artery. These flaps effectively cover the posterolateral portion of the defect [24]. The third Y-V flap is harvested from the mons pubis and advanced to cover the superior portion of the defect [21]. The combination of these flaps allows for efficient closure of extensive defects following vulvar cancer resection while minimizing the risk of complications. This technique is relatively simple to perform, does not require the precise dissection of perforating vessels, simplifies the surgical procedure, and reduces the risk of vascular injury [25]. A schematic visualization of the technique is shown in Figure 2.

### 2.3. V-Y Fasciocutaneous Advancement Flaps

The V-Y flap technique is one of the most commonly used methods in reconstructive surgery following vulvar cancer [26]. V-Y fasciocutaneous advancement flaps have been utilized in the reconstruction of defects following radical vulvectomy performed for the treatment of vulvar malignancies, such as squamous cell carcinoma. This technique allows for favorable aesthetic outcomes, rapid healing, and the preservation of sexual function [27]. The method is also applicable in the reconstruction of defects resulting from surgery for Paget’s disease, enabling effective closure after extensive tissue loss, minimizing the risk of complications, and facilitating the restoration of perineal function [22]. V-Y advancement flaps may also be used in the surgical management of perianal fistulas and defects associated with Crohn’s disease. This technique offers a viable option for treating complex fistulas and extensive perianal wounds in Crohn’s disease patients, improving healing outcomes and reducing recurrence rates [28].

**Figure 3:**
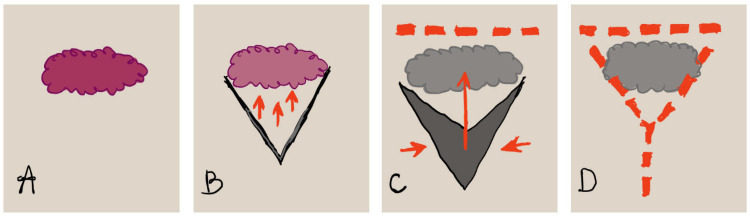
Visualization of V-Y fasciocutaneous advancement flaps: (**A**) The initial defect is marked in purple to outline the area requiring reconstruction. (**B**) A V-shaped incision is carefully made in the skin adjacent to the defect, followed by the undermining of the subcutaneous tissue beneath the “V” shape. (**C**) The V-shaped section of skin is then mobilized and advanced to cover the primary vulvar defect, with the medial edges approximated and closed. (**D**) The apex of the V-shaped flap is sutured in a linear fashion to complete the reconstruction.

This method involves making a V-shaped incision around the defect, mobilizing the flap along with subcutaneous tissue and blood supply, and advancing it forward to close the wound in a Y-shape. The technique leverages tissue adjacent to the defect [29]. A schematic visualization of this method is shown in Figure 3.

By relying on local perforators, it is possible to preserve vascularization and ensure an adequate blood supply, minimizing the risk of necrosis and promoting the flap’s viability [30]. An additional advantage of this method is the use of adjacent tissues, which share a similar structure and color, allowing for optimal tissue matching and favorable aesthetic outcomes. This technique also helps minimize the risk of wound dehiscence and infection [31].

### 2.4. Keystone Perforator Island Flaps

Keystone flaps are one of the reconstruction options following radical vulvectomy, offering rapid wound healing, satisfactory aesthetic outcomes, and functional preservation [32]. This technique enables the closure of large defects following resection of rectal and anal malignancies, while maintaining favorable surgical outcomes [33]. Keystone flaps are also an effective reconstructive technique for extensive defects resulting from the surgical management of necrotizing fasciitis of the perineum (Fournier’s gangrene), allowing for rapid and safe wound closure [34].

**Figure 4:**
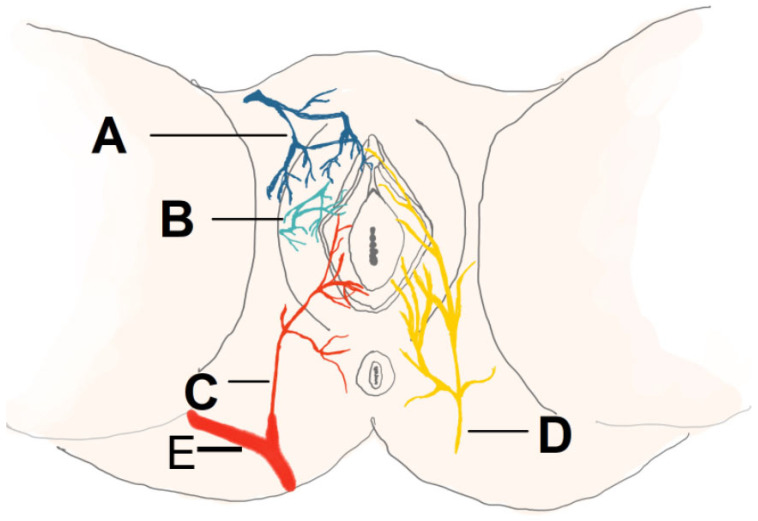
Arrangement of the arteries and pudendal nerves surrounding the vulva and perineal region: (**A**) The anterior labial artery, a branch of the external pudendal artery. (**B**) Cutaneous branches stemming from the obturator artery. (**C**) The posterior labial artery, originating from the internal pudendal artery. (**D**) The pudendal nerve. (**E**) Internal pudendal artery.

This technique provides coverage for perineal defects while maintaining flaps’ vascularization and elasticity. Reconstruction utilizes perforators from three arteries: the internal pudendal artery (mainly supplying the vulvar area), the inferior gluteal artery (supplying the gluteal area), and the deep femoral artery (supporting thigh tissues) [35]. A schematic illustration of the vascularization and innervation is shown in Figure 4.

The keystone flap is designed based on the identification of perforators using Doppler imaging. The flap is then incised and mobilized into a wedge or arch shape, allowing for natural adaptation to the defect without overburdening the blood vessels. The flexibility of the flap facilitates its transfer to the defect site without creating additional deficits at the donor site [7].

**Figure 5:**
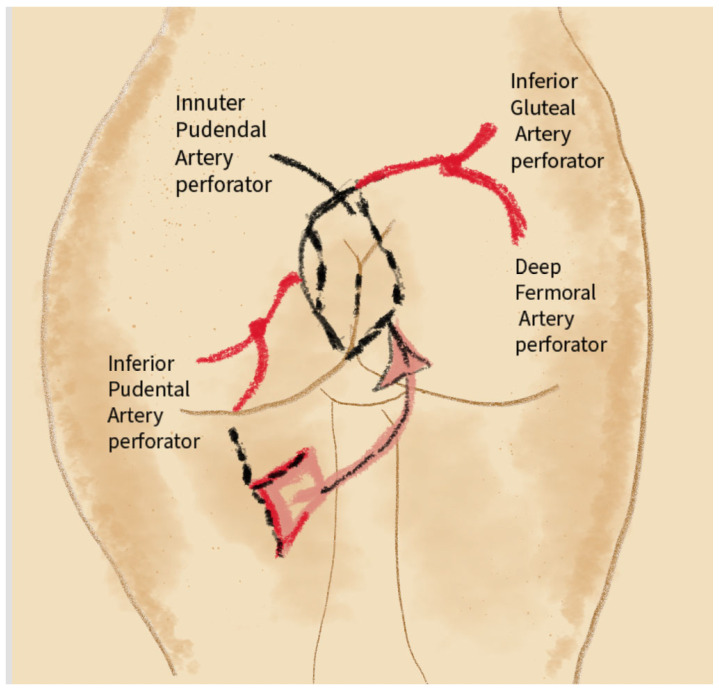
Keystone Perforator Island Flap.

The perforator-based “keystone” flap technique is a promising approach for soft tissue reconstruction, characterized by a high flap viability, a low risk of complications, and favorable aesthetic and functional outcomes [33]. A schematic visualization of this method is shown in Figure 5.

Illustration showing perineal reconstruction using the keystone flap technique (Keystone Perforator Island Flap), with marked perforators of the internal pudendal artery, inferior gluteal artery, and profunda femoris artery.

### 2.5. Anterolateral Thigh (ALT) Flaps

The anterolateral thigh (ALT) flap is effective in covering extensive soft tissue defects following resection surgeries for anal, rectal, and vulvar malignancies, as well as in patients after radiotherapy [36]. In cases of large tissue loss due to infections such as Fournier’s gangrene, the ALT flap provides reliable coverage and promotes wound healing [37]. In reconstructions following radical gynecologic surgeries, the ALT flap allows for restoration with adequate tissue volume and elasticity [36]. This technique also serves as an alternative to the Vertical Rectus Abdominis Myocutaneous (VRAM) flap when the latter is contraindicated [38]. The ALT flap may also be employed in cases of previous reconstructive surgery failure [39].

This reconstruction technique uses flaps from the anterolateral thigh, based on perforators from the lateral circumflex femoral artery. The flap is highly resistant to ischemic complications and can incorporate both musculocutaneous and fasciocutaneous components, making it versatile for reconstructing both superficial and deep perineal defects. The surgical procedure involves locating perforators in the anterolateral thigh region, harvesting the flap with an appropriate tissue volume, and transferring it to the reconstruction site using microvascular anastomosis [38]. The donor site is closed with minimal tension, avoiding significant functional deficits [40].

**Figure 6:**
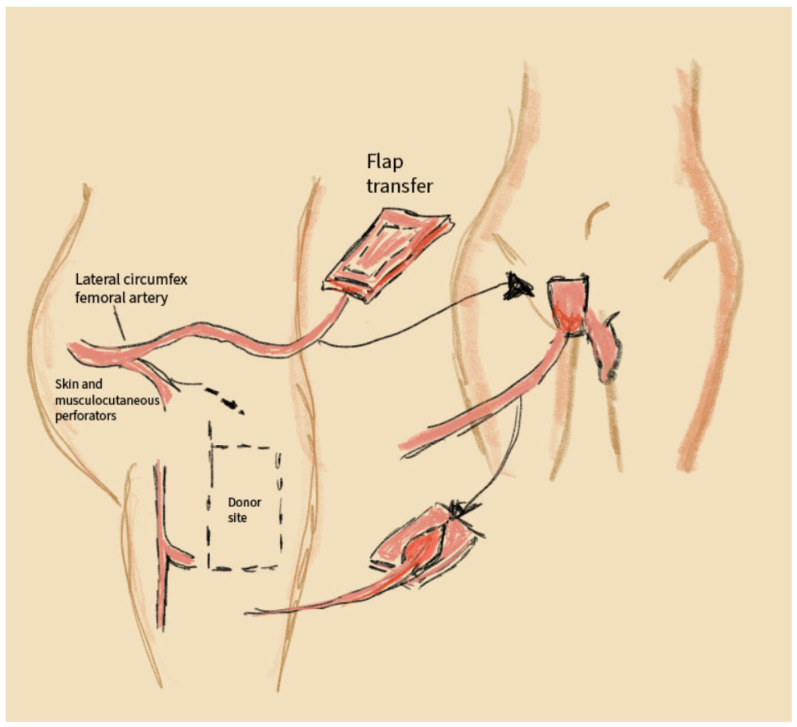
Anterolateral thigh flap (ALT flap): **Thigh anatomy:** Location of the lateral circumflex femoral artery. Cutaneous and musculocutaneous perforators arising from this artery. **Donor site:** A rectangular area marked on the anterolateral aspect of the thigh as the flap harvesting site. Possibility of harvesting only the skin and subcutaneous tissue or including muscle as well. **Flap transfer:** Transferring the flap to the perineal region. Lines indicating microvascular anastomosis with the recipient vascular system.

The anterolateral thigh flap is a valuable method in reconstructive surgery for vulvar and perineal cancer, offering an extensive flexibility of design, reliable vascularization, and minimal donor-site morbidity [41]. A schematic visualization of this method is shown in Figure 6.

### 2.6. VRAM Flap (Vertical Rectus Abdominis Myocutaneous Flap)

The Vertical Rectus Abdominis Myocutaneous (VRAM) flap is effective for closing perineal defects following abdominoperineal resection for anal or rectal cancer, reducing the risk of perineal wound complications [42]. The VRAM flap enables reconstruction following pelvic radiotherapy by providing well-vascularized, non-irradiated tissue that promotes healing [43]. This technique allows for the coverage of extensive defects following the excision of tumors infiltrating perineal structures. In reconstruction after total pelvic exenteration, the VRAM flap facilitates the restoration of the pelvic floor and perineal region [44]. The method is also used in reconstructive procedures following the excision of rectourethral or rectovaginal fistulas. It provides effective fistula closure and accelerates healing in the perineal region [43].

The VRAM flap relies on the vascular supply from the inferior epigastric artery. It involves harvesting rectus abdominis muscle tissue, along with skin and subcutaneous tissue, either as a pedicled or free flap. The procedure includes identifying and preparing the VRAM flap from the abdominal region, transferring it to the defect site, and establishing vascular connections (for free flaps) or directly closing the defect (for pedicled flaps). Donor-site closure is performed with minimal tension, often utilizing surgical mesh. This method is preferred for large and deep defects, particularly in cases requiring a durable tissue coverage [45].

**Figure 7:**
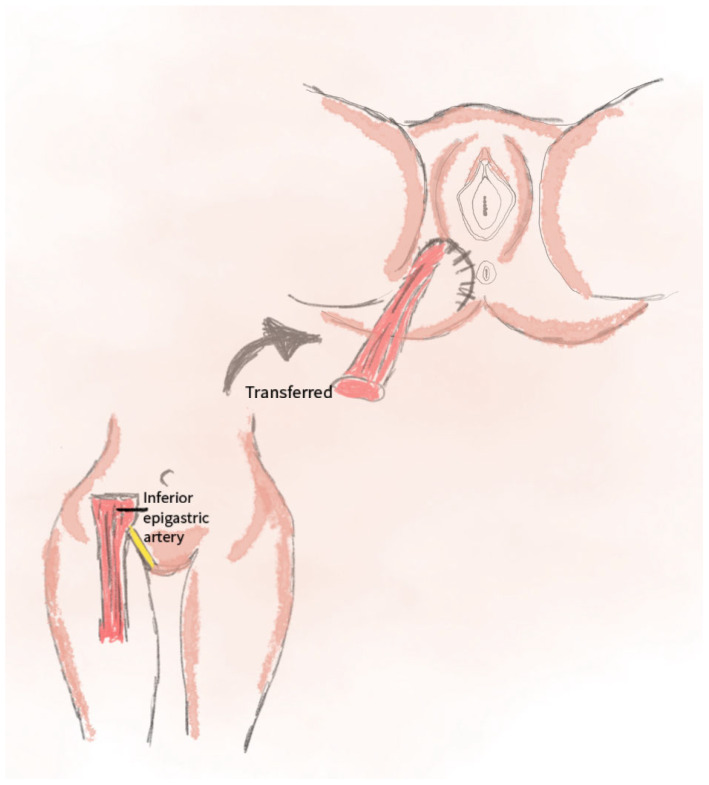
The illustration for the VRAM flap method includes the following: the location of the rectus abdominis muscle and its vascular supply; the transfer of the flap—the direction of the muscle translocation toward the defect area in the perineal/vulvar region.

This technique allows for the better restoration of superficial anatomy and enhances both aesthetic and functional outcomes [46]. Additionally, this method can be used for covering tissue defects that have previously undergone radiation therapy [47]. The VRAM flap is a valuable technique in vulvar and perineal reconstruction, providing the reliable vascularization and effective filling of extensive and deep defects [48]. A schematic visualization of this method is shown in Figure 7.

### 2.7. The Gracilis Flap

The gracilis flap is useful in tissue reconstruction following extensive oncologic procedures involving the vulva and vagina, providing an adequate tissue elasticity and volume [49]. The gracilis flap technique can also be employed for the closure of perineal fistulas, offering reliable coverage and supporting the healing process [50]. The gracilis flap is frequently utilized in reconstruction after major resection procedures, such as pelvic exenteration, delivering a sufficient tissue volume to fill the resulting defects [51]. This technique enables reconstruction in cases where other flaps are inadequate or contraindicated [52].

**Figure 8:**
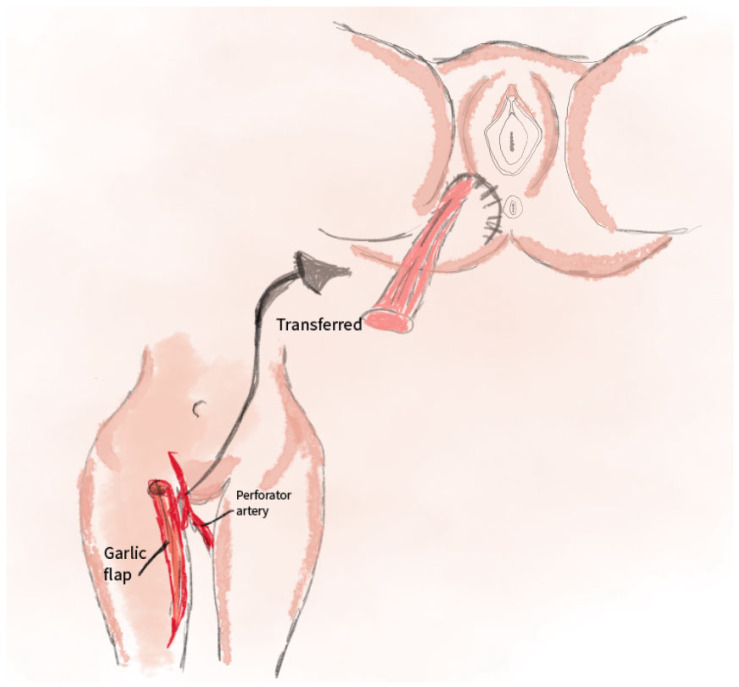
The illustration for the gracilis flap method includes the following: 1. The location of the gracilis muscle (m. gracilis) and the course of the perforating artery originating from the profunda femoris artery. 2. The transfer of the flap—the direction of the gracilis muscle translocation toward the defect area in the perineal/vulvar region.

The gracilis flap utilizes tissue from the gracilis muscle, supplied by perforating branches of the deep femoral artery. The muscle, with or without skin coverage, is harvested from the medial thigh. The surgical procedure involves dissecting the gracilis muscle, transferring the flap to the defect site, fitting the muscle, and optionally establishing vascular anastomosis. The donor site is closed with minimal scarring, making this method suitable for smaller defects. It minimizes functional deficits at the donor site and ensures a shorter recovery time [45]. The use of the gracilis muscle flap enables the successful surgical treatment of complex urethrorectal fistulas and can also be applied in the reconstruction of rectovaginal fistulas to achieve their closure [53]. The application of the gracilis muscle flap in perineal and vulvar reconstruction is a surgical method that allows for satisfactory restoration of tissue defects in these areas [54]. A schematic visualization of this method is shown in Figure 8.

### 2.8. PAP Perforator Flap Technique

The Profunda Artery Perforator (PAP) flap technique is utilized in the reconstruction of extensive defects in the vulvar and perineal regions, particularly following oncologic procedures such as abdominoperineal resection or vulvar cancer excision. This technique is especially valuable when harvesting conventional abdominal flaps is complicated due to prior surgical interventions [55]. The PAP flap is based on perforating vessels arising from the profunda femoris artery. These vessels traverse the adductor magnus muscle and provide vascular supply to the skin, subcutaneous tissue, and surrounding structures. The main perforators typically emerge approximately 2 cm posterior to the gracilis muscle and around 8 cm inferior to the inguinal crease, enabling safe and precise flap planning. The flap can be designed in horizontal, vertical, or oblique orientations, depending on the defect’s location and configuration. Preoperative imaging with CTA (Computed Tomographic Angiography) or MRA (Magnetic Resonance Angiography) is employed to accurately map the perforator vessels [56]. In perineal reconstruction following rectal and genital organ resection, the vertically oriented PAP flap (vPAP) has proven effective in closing large defects while providing an excellent soft tissue volume.

**Figure 9:**
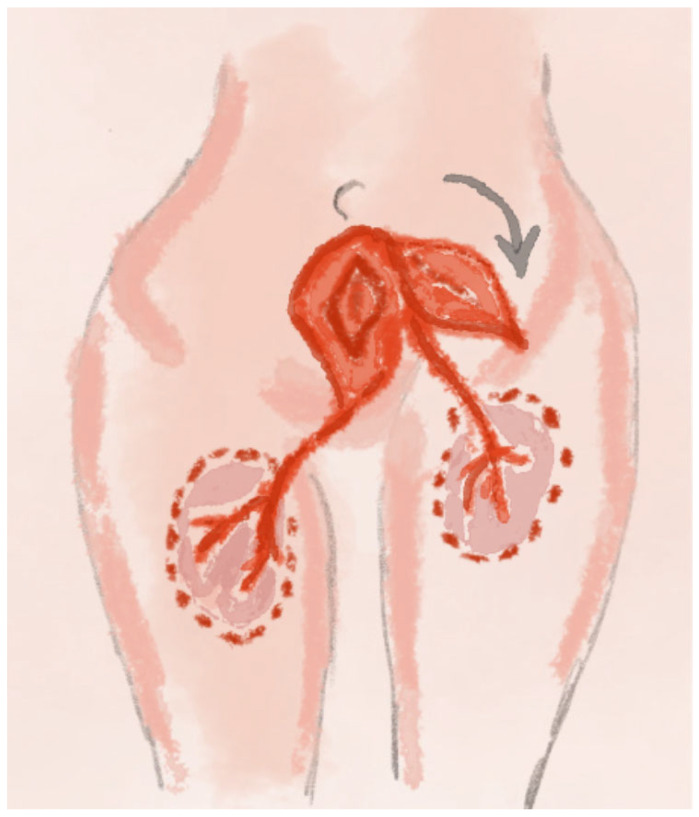
The illustration of the PAP perforator flap shows its vascular supply based on perforators arising from the profunda femoris artery, located below the inguinal crease.

A retrospective study involving the use of vPAP flaps in reconstructive surgery reported satisfactory clinical outcomes and a moderate risk of complications. Wound dehiscence occurred in 50% of patients, partial flap necrosis in 20% [57]. It is a good alternative when the abdominal donor site is unavailable. The PAP flap can also be combined with other flap techniques to manage extensive and complex perineal defects. One example includes the combination of a PAP flap with the gracilis muscle and bilateral Inferior Gluteal Artery Perforator (IGAP) flaps, which successfully restored a large perineal defect [58]. The PAP perforator flap technique is distinguished by its high degree of design flexibility and reduced donor site morbidity compared to musculocutaneous flaps. It offers high-quality tissue for reconstructive procedures while minimizing donor site complications. For these reasons, the PAP flap has become an increasingly preferred option in vulvar and perineal reconstruction [59]. A schematic visualization of the method is shown in Figure 9.

### 2.9. DIEP Perforator Flap Technique

**Figure 10:**
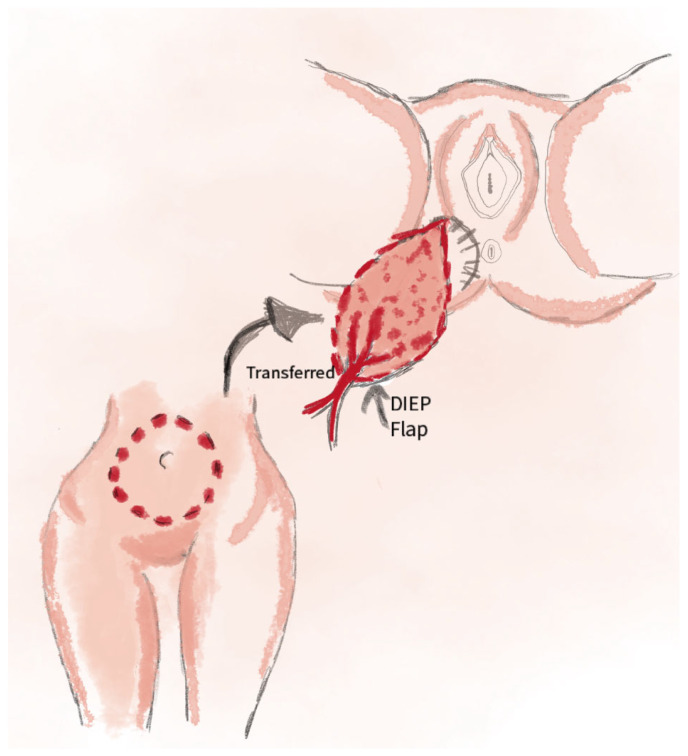
The illustration shows the use of the DIEP perforator flap, harvested from the lower abdominal region and based on perforators of the deep inferior epigastric artery, with preservation of the rectus abdominis muscle.

The Deep Inferior Epigastric Perforator (DIEP) flap technique is recommended for the reconstruction of extensive defects in the vulvar and perineal regions following oncologic procedures. This method enables effective coverage of the defect while maintaining minimal donor site morbidity [60]. This technique is also employed in vaginal reconstruction after pelvic exenteration. The flap is based on perforators of the deep inferior epigastric artery, offering adequate tissue coverage and preserving the functional integrity of the perineum [61]. The DIEP flap is utilized in surgical procedures following treatment for vulvar Paget’s disease. It allows for integration with other reconstructive techniques while minimizing the risk of complications [62]. This technique involves harvesting a skin-fat flap from the lower abdomen, based on perforators of the deep inferior epigastric artery, while sparing the rectus abdominis muscle. It provides reliable coverage for both superficial and deep defects, with minimal tension on wound edges and a low risk of postoperative complications [63]: 32% at the recipient site (dehiscence, necrosis); low at the donor site (3%) [63]. The DIEP technique is considered technically straightforward, as it does not require meticulous dissection of the perforating vessels. This simplifies the surgical procedure and significantly reduces the risk of vascular injury [64]. A schematic visualization of the method is shown in Figure 10.

### 2.10. Application of ICG Technique in Vulvar and Perineal Reconstruction

Fluorescence angiography using indocyanine green (ICG) is a modern method utilized for the intraoperative assessment of tissue perfusion. In vulvar and perineal reconstructions, it enables the real-time visualization of vascularization. This technique facilitates the precise tailoring of skin flaps and helps avoid ischemic areas during surgery [65]. The ICG technique involves the intravenous administration of indocyanine green, which binds to plasma proteins and emits fluorescence under near-infrared light, thereby allowing for the visualization of tissue vascularization. Its use in laparoscopic vaginoplasty or neovaginoplasty has enabled the intraoperative verification of sigmoid colon segments’ viability. This allows for modifications to the surgical plan in cases of insufficient vascularization [66]. In reconstructive procedures involving the gracilis muscle, ICG facilitates both a visual and quantitative analysis of perfusion, potentially reducing the risk of flap necrosis [67].

**Figure 11:**
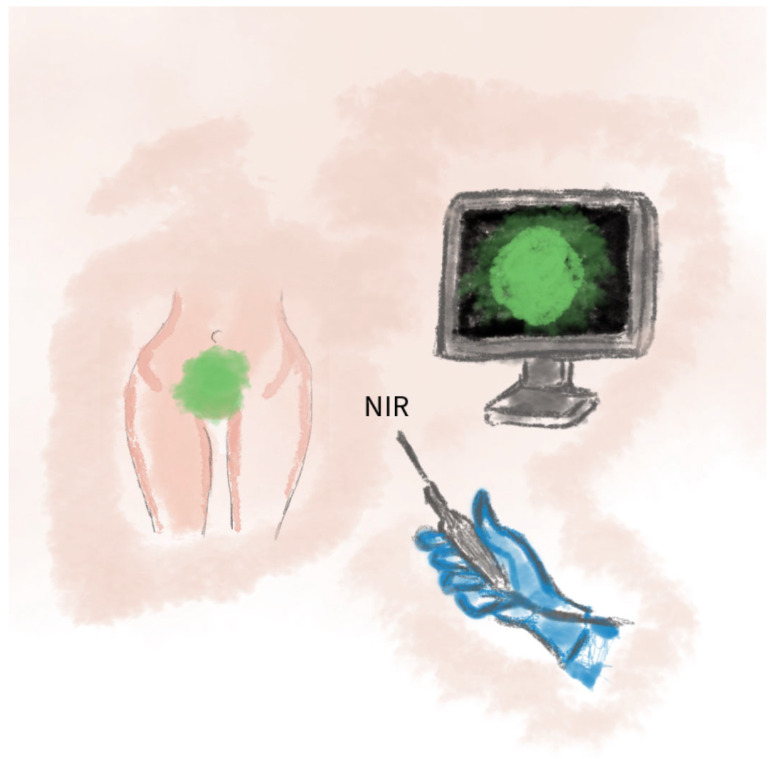
The illustration depicts the use of indocyanine green (ICG) fluorescence angiography during perineal reconstructive surgery.

The ICG technique can also be employed in the reconstruction of cloacal malformations and rectovaginal fistulas in pediatric surgery, thereby enhancing the safety of the procedure. This technique has demonstrated a 90% sensitivity in assessing flap viability. ICG imaging is recommended in reconstructions following extensive oncologic resections, in cases with a high risk of tissue necrosis, as well as in pediatric surgery [68]. Well-perfused areas emit fluorescence under near-infrared light. ICG imaging enables the intraoperative assessment of tissue viability and the precise tailoring of flaps, thereby reducing the risk of necrosis [69]. This provides increased safety by providing real-time perfusion assessments. A schematic visualization of the method is shown in Figure 11.

### 2.11. Robot-Assisted Flap Harvesting Technique

Robot-assisted flap harvesting techniques are increasingly utilized in reconstructive procedures for extensive perineal and vulvar defects, such as those following vulvar cancer resection, abdominoperineal excision of the rectum, or pelvic exenteration. The procedure involves the minimally invasive harvesting of a myocutaneous or fasciocutaneous flap using a robotic surgical system. This approach enables the precise dissection of vessels and soft tissues while minimizing donor site trauma [70].

**Figure 12:**
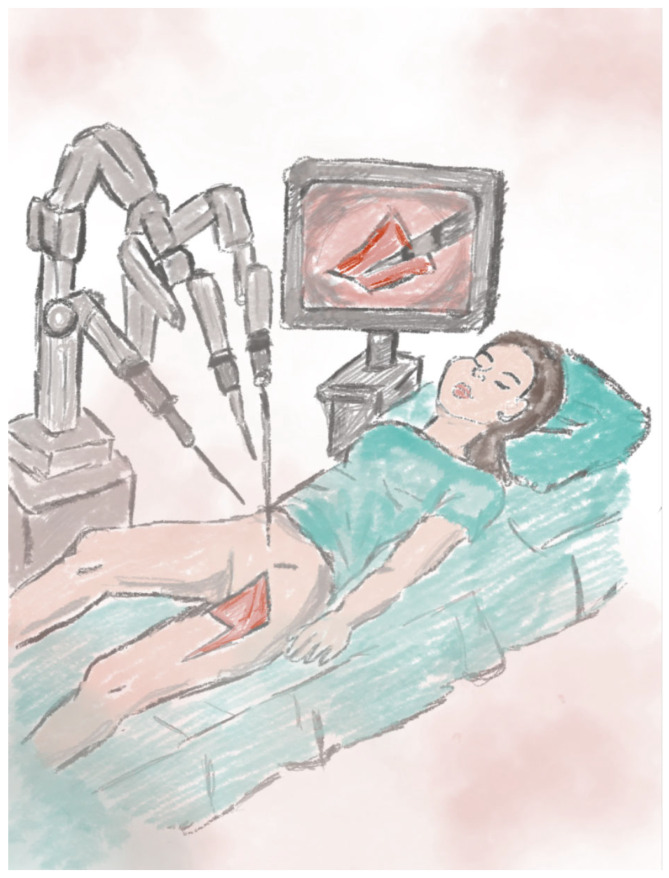
The illustration shows the robot-assisted harvesting of a myocutaneous flap during perineal reconstructive surgery.

This technique can be applied in reconstruction following abdominoperineal resection in oncological patients. The gracilis muscle flap is harvested with robotic assistance, allowing for a reduced hospitalization time and a lower rate of perioperative complications [71]. It is also used in the reconstruction of complex pelvic defects following total vaginectomy and proctectomy. A robotically harvested gracilis flap has been employed to reinforce the neoseptum between the bowel and the neovagina, preventing fistula-related complications and improving perineal aesthetics [72]. Combining robot-assisted flap harvesting with conventional reconstructive techniques enables complete defect coverage while preserving perineal function and aesthetics. The robotic system enhances the visualization of anatomical structures, allows for precise incisions, and minimizes tissue damage [73,74]. A schematic visualization of the method is shown in Figure 12.

## 3. Surgical Outcomes of Vulvar and Perineal Reconstruction: A Comparative Analysis of Techniques

Reconstructive surgery of the vulva and perineum following extensive oncologic excisions necessitates a tailored surgical approach. The choice of technique should be based on the size and depth of the defect, local tissue conditions, and the patient’s overall health. Among the techniques analyzed, gluteal fold flaps stand out for their ability to restore sensory function and achieve a natural appearance, with minimal complications reported in the literature [16]. The triple flap method has proven effective for addressing large, complex defects. By combining flaps from the mons pubis and gluteal region, this technique promotes tension-free wound closure, reduces healing complications, and delivers both functional and aesthetic benefits [21]. In smaller defects, V-Y fasciocutaneous advancement flaps offer a straightforward solution, providing sufficient tissue mobility with minimal donor-site morbidity. Although most patients recover well, some experience mild postoperative discomfort during sitting [29]. Keystone flaps, based on multiple perforators, provide a reliable option for moderate-to-large perineal defects. They are praised for their simplicity, flexibility, and favorable healing outcomes with a low rate of necrosis [7]. Anterolateral thigh (ALT) flaps are particularly useful in covering extensive defects. Their robust vascular supply allows for reliable tissue integration, although the need for microsurgical expertise can limit their accessibility [38]. In contrast, VRAM flaps offer substantial soft-tissue bulk and are well-suited to fill deep or irradiated wounds. However, complications at the donor site, such as abdominal wall hernias, remain a concern [45]. The gracilis muscle flap is often employed for smaller perineal defects due to its ease of harvesting and minimal donor site issues. Nonetheless, its relatively limited size and vascularity may restrict its application in larger reconstructions [54]. Across the various techniques, the postoperative restoration of urinary and fecal continence was consistently achievable. The most favorable outcomes in terms of sexual function were observed with procedures that preserved or restored innervation—especially those using sensate gluteal flaps. Ultimately, reconstructive strategy should be individualized to maximize healing, restore anatomical function, and enhance quality of life. A comparison of perineal and vulvar reconstruction methods is shown in Table 1.

## 4. Challenges and Possible Complications of Reconstruction Methods

The complications and risks associated with vulvar and perineal reconstruction represent a pivotal component in the evaluation of surgical procedures, with regard to both efficacy and safety. In the context of advanced perineal cancer, frequently accompanied by reconstruction, the complication rate is notably high, ranging from 25% to 60%. This has a substantial impact on patients’ quality of life. The anatomical complexity of the region, coupled with the proximity of vital structures such as blood vessels, nerves, and internal organs, causes each surgical method to be associated with a unique set of risks and potential complications [45].

A significant contributing factor to perineal wound complications is the presence of dead space (empty pelvic syndrome) within the pelvic cavity subsequent to oncological resection, and the difficulties associated with filling this area during reconstructive procedures [75]. Furthermore, factors such as a history of radiotherapy, smoking, and skin laxity in the inner thigh, which is more prevalent in older, slim patients without obesity, and the asymmetry of the defect, are also important aspects to consider [15,76].

### 4.1. Complications Related to the Proximity of Blood Vessels

The vulva and perineum are areas with intense vascularization, which unfortunately increases the risk of bleeding during reconstruction. The proximity of large vessels, such as the internal vulvar artery, poses a potential risk of serious hemorrhage if they are damaged [77]. Furthermore, the abundant blood supply in these regions can lead to the formation of postoperative hematomas, which can prolong the healing process, increase the risk of infection, and even result in pressure on surrounding tissues, potentially leading to necrosis [78].

### 4.2. Complications Depending on the Method of Reconstruction Used

The nature of complications and their associated risks during vulvovaginal reconstruction are predominantly influenced by the surgical method employed. Anatomical variations, vascularization, and the extent of tissues utilized during the procedure can influence the specificity of the risks. Each technique carries unique risks, including but not limited to flap ischemia, the dysfunction of adjacent structures, and infection. Consequently, these factors must be meticulously evaluated during the surgical planning process. The optimal surgical technique should provide the requisite amount of tissue to cover the defect, ensuring the shortest possible healing time and improving the patient’s quality of life while minimizing the occurrence of complications [21].

### 4.3. Sensate Gluteal Fold Flaps

The Sensate Gluteal Fold Flaps technique is a surgical procedure that involves the use of tissue from the gluteal fold to reconstruct defects in the perineum and vulva. It is characterized by a low risk of complications and good functional results. Due to its wide coverage, it can be used even in extensive vulvar defects [52]. However, the potential disadvantages of this technique include the discomfort felt by patients while sitting. Complications have occurred in 13.6% of patients, including donor site dehiscence and recipient site infections, which were managed conservatively [16]. The most significant limitation of this method is the possibility of an insufficient flap length, which may prevent full closure of the defect over the pubic conjunctiva [26].

### 4.4. V-Y Fasciocutaneous Advancement Flaps

This method is considered less invasive because it involves only a local tissue manipulation. This approach is associated with a reduced risk of complications arising from the donor site. A study of 30 patients who underwent vulvar reconstructions with a total of 59 flaps showed that minor complications were reported in 23% of patients (14% of flaps), with wound dehiscence being the main complication [31]. A 2018 study yielded similar results, with complications occurring in 33% of patients and no cases of flap necrosis or recurrence [29]. A further study revealed that patients who underwent the V-Y flap approach had a reduced average length of hospitalization and a lower complication rate compared to those who underwent radical surgery alone (a difference of 11% to 40%) [30]. However, it is important to note that this method is associated with postoperative discomfort and pain in the sitting position [79]. A further study revealed that four patients in the control group who underwent the procedure experienced tissue necrosis, suggesting that the size of the tumor may be a contributing factor. The study also observed that the complication rate was lower in women with a lesion measuring >4 cm undergoing vulvar–perineal reconstruction. Wound dehiscence occurred in 10.3% of patients in a cohort group and in 40% of the control group. Stenosis and urinary tract infection occurred in both study groups, with prevalence rates of 7% and 17%, respectively. Deep vein thrombosis occurred in eight patients in the entire study [30]. Complicated wound healing was observed in 33% of patients, resulting from wound dehiscence or scar damage, and infections were found in 15% of patients, 50% of whom had a urinary tract infection, and the remaining 50% had postoperative fever. The black race was associated with an increased incidence of infectious complications compared to the white race. Otherwise, patients with diabetes or HIV infection were more likely to succumb to infections compared to healthy patients. However, the underlying reasons for these observed disparities in risk remain to be elucidated. No additional demographic or surgical variables were identified as contributors to wound or scar complications post-surgery. Due to the limited number of patients and complications included in the study, the findings require further investigation with a larger sample size [29]. The probability of sensory hypersensitivity in the operated area and the occurrence of lymphedema, which can cause discomfort, is increased. The method is characterized by rapid healing; infectious complications or damage to the marginal flap are rare, and patients experience minimal discomfort when sitting and walking [80].

### 4.5. Triple Flap Technique (Triple V-Y Flap)

The Triple Flap Technique is a valuable method of reconstruction, especially in cases of extensive defects where the use of a single flap would not provide adequate coverage of the defect. One possible complication is wound dehiscence, but the use of negative pressure therapy has been shown to significantly reduce this risk. No major complications were reported [21]. The technique has provided excellent aesthetic outcomes, minimal donor site morbidity, and no systemic complications.

### 4.6. Keystone Flaps (Keystone Perforator Island Flaps)

This technique rarely leads to complications, such as complete or partial flap loss, with the most common being partial wound dehiscence and incontinence. Subjects reported an enhancement in both self-esteem and quality of life. No issues with micturition or defecation were reported, and pain was reported as moderate [81]. The survival rate of the transplanted flap was estimated at 99.6%. Postoperative swelling and pain were minimal, with the most likely cause of the pain being temporary tissue denervation [82]. Once the nerves were supplied and the healing process was complete, the pain subsided, with only mild sensory disturbances in some. However, poor skin flaccidity may limit the use of this method. This method allows the early activation of the patient, which avoids postoperative swelling of the limbs. The most common complications include wound dehiscence and partial necrosis of the flap [83]. Using this technique, the return of normal sensory function can take several months [84]. In one patient there was a partial loss of the flap [85].

### 4.7. Anterolateral Thigh Flap (ALT Flap)

A study of patients who underwent APR reconstruction or pelvic evisceration defects with ALT flaps revealed that the most common complication was partial perineal wound dehiscence. Complications were observed in 39.1% of patients: wound dehiscence in 26.1%, major complications such as sacral osteomyelitis in 8.7%. All wounds eventually healed properly, except a case of sacral wound dehiscence and osteomyelitis [38]. The disadvantage of this surgical technique is the change of the patient’s position during the procedure [26]. Despite the relatively high complication rate, the ALT flap was effective for closing large defects with minimal donor site issues [38].

### 4.8. Gracilis Flap

The gracilis flap is one of the most versatile techniques for perineal reconstruction, offering a relatively uncomplicated procedure with a satisfactory success rate. There were fewer donor site complications compared to VRAM; 51% of patients resumed sexual activity, indicating a good post-reconstruction quality of life [14]. Minor complications occurred in 25% of patients, while major complications were reported in 17.5% [45]: Donor site complications (16%), wound dehiscence (27.6%) (higher than VRAM), marginal flap necrosis (7%), cellulitis (9.3%). Obesity and smoking have been identified as factors that significantly increase the risk of adverse sequelae. The primary risk associated with the utilization of the gracilis flap is its limited vascularization, which can result in wound dehiscence and, in extreme cases, flap necrosis [45].

### 4.9. VRAM

VRAM is regarded as a more reliable technique in comparison to the gracilis flap, due to its abundant tissue vascularization and greater tissue volume, resulting in a reduced incidence of complications such as flap necrosis [45]. However, it should be noted that VRAM is associated with more invasive tissue harvesting and potential weakening of the abdominal shell, which is more likely to cause abdominal hernias. The overall donor site complication rate was 57.6% (the highest among all techniques). Hernias occurred in 7.2%. Despite the high donor site complication rate, VRAM is considered highly effective for large defects due to its volume and reliability [45].

### 4.10. PAP Flap (Profunda Artery Perforator Flap)

The Profunda Artery Perforator (PAP) flap is a reliable option for perineal reconstruction, particularly in cases where abdominal tissue cannot be utilized. A retrospective study of 15 patients revealed an overall complication rate of 53.3%, with the most prevalent being wound dehiscence at both the donor and recipient sites (20% each) [57]. In a separate cohort of 10 patients following abdominoperineal resection (APR), major complications included venous congestion requiring a return to the operating room (20%), partial flap loss (20%), wound dehiscence (50%), the formation of abscesses (30%), and the development of fistula requiring surgical intervention (20%) [86]. A further study, which involved a total of six patients who underwent pelvic reconstruction using a combination of PAP and bilateral gracilis flaps, reported wound dehiscence in four cases and one instance of postoperative bleeding requiring embolization. The remaining flaps survived without complications [59]. It is important to note that the reported complications are based on studies involving relatively small patient cohorts, which limits the generalizability of the outcomes.

### 4.11. DIEP Flap (Deep Inferior Epigastric Perforator Flap)

The Deep Inferior Epigastric Perforator (DIEP) flap is an effective option for perineal reconstruction, especially in patients following extensive pelvic cancer resections. A retrospective study involving 34 patients who underwent perineal reconstruction with the DIEP flap reported a low rate of donor site complications (3%) and recipient site complications in 32% of patients, including infection, partial or complete flap loss, wound dehiscence, hematomas, and fistulas [63]. This method also necessitates a protracted operative time and an ideal patient anatomy, factors that can prove to be restrictive in oncological cases.

### 4.12. ICG-Guided Reconstruction (Indocyanine Green Fluoroscence Imaging)

Indocyanine green (ICG) fluorescence angiography is a useful adjunct in perineal reconstruction, allowing the real-time assessment of flap perfusion. It facilitates the intraoperative modification of flap designs and contributes to a reduction in complications, such as ischemia and necrosis, particularly when employed in conjunction with flaps including the gracilis or ALT. Although not a reconstructive method in itself, it has been demonstrated to enhance surgical safety. The utilization of this apparatus necessitates the employment of specialized equipment and the acquisition of advanced training, with rare complications including allergic reactions. The extant evidence base is, at present, limited to small case series and case reports. This situation highlights the necessity for larger studies to be conducted [69,87].

### 4.13. Robot-Assisted Flap Harvesting

Robotic techniques have been demonstrated to enhance the precision of flap harvesting, particularly in complex perineal reconstructions. The minimally invasive nature of these procedures has been demonstrated to reduce the incidence of donor site trauma and postoperative pain. This approach has demonstrated efficacy in reducing the duration of hospitalization and enhancing cosmetic outcomes. Nevertheless, the primary challenges confronting this field are the limited accessibility of the procedure, the substantial financial outlay required, and the necessity of a surgical team with a highly specialized training. Although perineal wound complications are uncommon, isolated cases of abdominal pain or abscess have been documented [70]. A retrospective study comparing robotic and traditional methods in 36 patients demonstrated comparable operative times and major complication rates, with a trend toward fewer minor complications in the robotic group (31% vs. 55%) [73].

### 4.14. Anatomical Proximity and Risk of Complications

The proximity of anatomical structures, such as the urethra, rectum, and vulvar nerves, has been demonstrated to increase the risk of damage to these structures during reconstructive procedures [88]. Damage to the urethra, for instance, has been shown to result in urinary problems, while a breach of the rectum can lead to fecal incontinence [89]. Furthermore, damage to the vulvar nerves can result in sensory disturbances and pain in the perineal area [90].

## 5. The Role of Mental Health in Gynecological Treatment

The psychological condition of patients undergoing gynecological surgery, particularly in oncological contexts, plays a fundamental role in both short- and long-term treatment outcomes. In the case of vulvar cancer, where extensive resections often lead to significant anatomical and functional loss, reconstruction is not only a physical necessity but also a psychological imperative.

Studies have consistently demonstrated that preoperative mental health significantly influences recovery, particularly in surgeries involving intimate areas such as the vulva and perineum [91]. For example, the use of Sensate Gluteal Fold Flaps, aside from offering good anatomical and aesthetic outcomes, is particularly valuable for patients with pre-existing psychological vulnerabilities, as the preservation of sensation in the reconstructed area contributes positively to self-esteem, body image, and sexual function [16]. Similarly, the Triple Flap Technique, often used in extensive reconstructions, may be psychologically demanding due to the complexity and duration of the procedure. Preoperative counseling and expectation management are crucial in these cases to reduce anxiety and improve postoperative satisfaction [21]. Preoperative psychological assessments and patient education can reduce adverse outcomes such as anxiety, depression, and body image disturbances [92]. The V-Y fasciocutaneous advancement flap, while less invasive and technically simpler, can result in discomfort when sitting and may alter body image perceptions. In such cases, postoperative psychological support is beneficial to address issues such as frustration, altered self-perception, or concerns about femininity and sexuality [29]. Similarly, the choice of ALT or VRAM flaps in cases of extensive defects—while technically effective—requires a discussion with patients about visible scarring, functional limitations at the donor site, and longer recovery times. These factors can contribute to psychological distress if not adequately addressed before surgery [38,45].

The necessity for an integrated approach to patient care—addressing both physical and psychological health—is strongly emphasized, and psychological screening should be integrated into preoperative assessment protocols for vulvar cancer patients [92]. Research has indicated a correlation between the desire for maternity and the level of tension towards the intervention. The aspiration for childbirth was associated with elevated levels of tension in relation to the intervention. These findings indicate that variations in psychological distress can be ascribed to a range of factors, including indications, methodologies, typologies of intervention, and the nature of the provided information [93]. As demonstrated in the following review, younger women, highly educated women, and women residing alone are more prone to experience psychological distress. Factors such as the desire for motherhood, fear of sexual dysfunction, and previous trauma significantly influence postoperative well-being [94].

The impact of surgical treatments for vulvar cancer on quality of life and sexual function has been the subject of numerous studies, which have utilized validated questionnaires to gather empirical data. The Female Sexual Function Index (FSFI) and the Short Form-12 (SF-12) have been utilized in scientific studies to evaluate postoperative outcomes. The results demonstrated that lymphadenectomy had a detrimental effect on sexual function, while age and the extent of surgery did not show a statistically significant influence [95]. Another study utilized the EORTC QLQ-C30 and QLQ-C29 questionnaires to evaluate quality of life in patients following perineal reconstruction with a Vertical Rectus Abdominis Myocutaneous (VRAM) flap. Patients who underwent reconstruction reported reduced fatigue and fewer skin-related complaints compared to those who did not undergo reconstruction. However, a higher prevalence of abdominal wall hernias was observed in the reconstruction group. While the present study concentrated on rectal cancer, it emphasizes the significance of VRAM flaps in perineal reconstruction for enhancing long-term quality of life, including in vulvar oncology [96]. Furthermore, the Functional Assessment of Cancer Therapy—Vulvar (FACT-V) questionnaire has been utilized to evaluate quality of life in patients treated for vulvar cancer, including those undergoing reconstructive procedures. The tool effectively captured emotional, physical, and functional impairments, highlighting the significant impact of surgical and reconstructive interventions on daily functioning [97].

Ultimately, an interdisciplinary approach that includes surgeons, oncologists, and mental health professionals is necessary. Psychological care offered both before and after surgery should be considered an essential element of treatment, particularly in procedures involving the reconstruction of the vulva and perineum, where the outcomes strongly affect not only the physical but also emotional and relational dimensions of a patient’s life [92].

## 6. Limitations

Based on the literature reviewed in this article, it should be noted that most of the available evidence on the efficacy of vulvar and perineal reconstructive techniques is derived from case reports, retrospective studies, and investigations conducted on relatively small patient cohorts. There is a clear lack of randomized controlled trials directly comparing various reconstructive methods, which limits the development of standardized clinical guidelines. Moreover, the heterogeneity of study populations, along with differences in oncologic indications and prior treatments (such as radiotherapy), may significantly impact the outcomes and their interpretation. Therefore, high-quality prospective studies are needed to enable an objective evaluation of the effectiveness and safety of individual reconstructive techniques.

## 7. Conclusions and Summary

Recent years have seen significant advances in the field of vulvar and perineal reconstruction. Current clinical data and surgical experience underscore the necessity of individualized strategies in vulvar and perineal reconstruction. These strategies should be tailored to the anatomical and oncological characteristics of the defect, the patient’s overall health status, previous treatments (including radiotherapy), psychosexual needs, and psychosocial determinants. A holistic approach is gaining increasing importance, prioritizing postoperative quality of life over a strictly oncologic focus.

In this context, it must be emphasized that the selection of the most appropriate reconstructive method should be individualized, considering the extent of the defect, the patient’s general condition, her personal preferences, and the surgical team’s expertise. In cases involving extensive tissue loss or prior exposure to radiotherapy, the use of well-vascularized myocutaneous flaps (e.g., VRAM, ALT) is recommended, as they provide a sufficient soft tissue volume and reduce the risk of complications such as flap necrosis and infections. For less advanced defects, local flap techniques (e.g., V-Y fasciocutaneous advancement flaps) remain a safe and effective option, provided there is no excessive tissue tension and no anticipated need for adjuvant therapy. Recent observations also highlight the beneficial impact of sensate or neurotized flap techniques (e.g., gluteal fold flaps) on sexual function and proprioception within the reconstructed region. Therefore, surgical planning should not only address morphological restoration but also the potential for reinnervation, which plays a crucial role in the patient’s long-term comfort and psychosexual rehabilitation. From a clinical perspective, close interdisciplinary collaboration is essential, encompassing gynecologic oncologists, reconstructive surgeons, psycho-oncologists, and physiotherapists. Routine psychological assessment prior to reconstruction and access to perioperative psychological support should be considered standard components of care.

Looking ahead, there is a strong rationale for developing standardized surgical decision-making algorithms that integrate anatomical, oncological, and psychosexual variables. Such frameworks will help optimize therapeutic outcomes and minimize the risk of complications in this complex domain of reconstructive surgery.

## Figures and Tables

**Table 1:** Advantages and disadvantages of surgical methods in perineal and vulvar reconstruction.

Methods	Advantages	Disadvantages
**Sensate Gluteal Fold Flaps [16].**	- Retained innervation in the flaps enables the restoration of sensory function. - The scar is located within the natural gluteal fold, improving aesthetic outcomes. - Minimized risk of necrosis due to the well-matched tissue profile for the perineal region.	- Not suitable for covering large defects. - Time-consuming and labor-intensive procedure.
**Triple Flap Technique [21].**	- Allows complex reconstruction of large defects while maintaining function and aesthetics, without the need for distant flaps. - Combining flaps facilitates the precise adaptation of tissues to the defect. - Minimizes the risk of wound dehiscence.	- Prolonged operative time. - Increased risk of flap necrosis due to the complexity of the procedure.
**V-Y Fasciocutaneous Advancement Flap [29].**	- Relatively simple technique requiring minimal donor-site intervention. - Ideal for smaller and superficial defects. - Minimal risk of donor-site complications. - Shorter operative and recovery times compared to other methods. - Maintains the anatomical and functional integrity of adjacent tissues.	- Restricted use for deep and large defects. - Inferior aesthetic results compared to more advanced methods.
**Keystone Perforator Island Flaps [7].**	- Suitable for medium and large defects. - Faster procedure time with a low risk of flap necrosis. - Good vascularization provided by perforators from three arteries. - No need to create a separate donor site.	- Less effective for defects requiring a significant tissue volume. - Requires operator expertise in utilizing perforator flaps.
**ALT Flap [38].**	- Provides a large amount of tissue, making it an ideal method for very large defects. - Minimal functional deficit at the donor site. - Can be used as either a free or pedicled flap.	- Longer surgical duration with microvascular requirements. - Requires an experienced operator.
**VRAM [45].**	- Stable blood supply. - Capable of covering large and deep defects. - Provides good functional outcomes.	- Risk of complications at the donor site, such as abdominal hernia. - Potential impact on abdominal muscle functionality.
**Gracilis Flap [45].**	- Simpler technique, suitable for smaller defects. - Lower risk of donor-site complications. - Shorter operative time.	- Restricted tissue volume for reconstruction.
**PAP Flap [57].**	- Minimally invasive. - Flexible flap design. - Suitable for extensive tissue defects.	- Requires precise vascular imaging - Limited donor tissue volume in slender patients
**DIEP Flap [61].**	- Preserves the rectus abdominis muscle. - Favorable aesthetic outcomes. - Covers large and deep defects.	- Prolonged operative time - Requires favorable abdominal anatomy
**ICG Angiography [65].**	- Real-time tissue perfusion assessment. - Reduces the risk of flap necrosis.	- Requires specialized equipment - Risk of hypersensitivity reactions to indocyanine green
**Robotic-Assisted Flap Harvesting [70].**	- High precision with minimal donor site trauma. - Satisfactory aesthetic outcomes. - Shorter hospitalization.	- High cost and limited availability - Requires an experienced surgical team
